# The Present Status of the Pessary in Gynecological Practice

**Published:** 1873-01

**Authors:** Philip Adolphus

**Affiliations:** 318 West Madison St.; Chicago


ARTICLE IV.—*The Present Status of the Pessary in Gynecological Practice.* By PHILIP ADOLPHUS, M.D., Chicago.

In classical treatises the pessary is the remedy *par excellence*, in flexions, versions, prolapse; in cases of irritable uterus, etc.

It is the aim of the writer to indicate its true sphere of usefulness, and to limit its indiscriminate use, without acquiescing exclusively in mere theories, whether inflammatory or mechanical.

The moral weight of the French school, led by Velpeau, imposed a theory and practice entirely mechanical, which, since 1854, has dominated over the medical world, and the result of which may still be seen in the thronged show-cases of the instrument vender, where pessaries of every shape, size and material are on exhibition.

Do uterine displacements, of themselves, produce any pathological state of the organ? Or, are these displacements caused and maintained by some complication, such as ulceration, engorgement, granulation, etc.?

Again, are the symptoms which accompany displacements, the results of nervous irritation, inflammatory congestion of the womb, ovarian complications, or disturbance of the ganglionic system?

No mere theory of any man or school, however eminent, can determine this. Observation alone, conducted by practical men, not biased by any theory, can elicit a practice which will maintain the golden medium of safety.

In order to determine in what affections the pessary should be advantageously used, the writer will avail himself of the method of exclusion, and state where it ought not to be employed.

It should be interdicted in diseases superinduced by morbid menstruation, whether the menstrual flow is increased, deficient, or ceases entirely.

According to Tilt, "the ovary ranks above the uterus; the womb is the appendage of the ovaries, as the bladder is that of the kidneys. It is the ovary which calls the uterus into action, imparting to it a stimulus which is either healthy or morbid, periodical or continuous."

Therefore the inciting cause of menstruation resides in the ovary; not the womb. The periodic turgescence of the ovaries is admitted by all. How easily, then, may not an exciting cause, such as sexual intercourse, cold, emotional shocks, stem pessaries, caustics to the neck of the womb, instrumental measures, injections, etc., transform the only physiological process in the body which is attended by nervous disturbance, into a series of *pathological phenomena*, which in their various manifestations will awaken, by means of the sympathetic nerves and their ganglia, the *solar plexus*, which, through the instrumentality of the spinal nerves, connecting with the ganglia, transmits the morbid sensations to the brain.

For the sympathetic system, besides other functions, according to Bichat, Winslow, Brown-Sequard, and others, "constantly reacts upon the cerebro-spinal system; in health imparting a sense of strength, without any sensation referable to the organs of vegetative life; but should they be diseased, then the ganglionic nervous system convinces the brain that it is intimately associated with a kidney, a liver, or a womb."

Hence the various symptoms of physiological menstruation, viz., ganglionic cerebral and spinal; the sanguineous mucous, and gastro-intestinal discharges; its influence on the cutaneous, and on the urinary surfaces, and urinary deposits, are intensified by becoming pathological. Thus we have these symptoms imputed by authors to various affections of the uterus, when in fact they originate in the ovaries.

In all these affections the condition of the pelvic organs should be considered; not merely the uterus, but the ovaries and fallopian tubes, are more or less morbidly influenced; therefore, the introduction of any foreign substance tends to produce cellulitis, pelvi-peritonitis, and hæmatocele. Indeed, the writer asserts that uterine diseases and uterine pathology should be entitled, respectively, ovario-uterine diseases, and ovario-uterine pathology. Were this opinion generally acknowledged by physicians, our practice would accord with correct anatomical and physiological principles.

Therefore, it is to be recommended, that, before commencing treatment in chronic diseases of the pelvic organs, complicating affections of their peritoneal coverings and the cellular tissue be excluded. Serious consequences are often caused by pessaries and other procedures, when milder expedients might have avoided any risk.

Pessaries are contra-indicated in cases of flexions where the womb is retained by cellular tissue, due to peritoneal exudations. It is here impossible to restore the organ without violently rupturing these adhesions.

In other cases where the uterus is movable, the intra-uterine pessary no doubt corrects the faulty position and retains the organ, yet its use is so dangerous, that it is universally condemned by the profession, and has consequently fallen into disuse.

Versions, as such, do not require treatment; when, however, the displaced womb is diseased, this becomes essential. Let the congestion and sub-acute inflammation that attends uterine versions be eliminated, and the pessary would be seldom required. The ever recurring ovarian irritation and uterine congestion is, however, ultimately extinguished by cessation of menstruation; and many women, at that period, with versions, lead without inconvenience an active life.

No pessary should be introduced when ovarian and metritic inflammation is present, or after the occurrence of the third month of pregnancy.

Lastly: cases where the vagina is delicately organized, making it intolerant of a hard substance, or where a prolapsed or inflamed ovary is present, likewise forbid its introduction.

Having eliminated those pathological states in which the application of the pessary is injurious, the writer will proceed to enumerate the conditions in which its use is advantageous.

Abortions are sometimes caused by a retroverted state of the uterus; this accident is obviated by wearing a pessary up to the period of quickening, for then the organ rises out of the pelvis.

When flexions of the uterus cause sterility, by preventing the entrance of semen into its cavity, the wearing of a pessary during intercourse is often followed by the happiest results.

Exceptionally, we find patients where the introduction of a pessary dissipates constitutional disturbances, irritation of the rectum and bladder; and enables immediate walking, without the slightest cognizance by the patient of the presence of the pessary. Every busy practitioner has met such cases.

However, the general average of cases teaches us, that with the exception of prolapse, uterine deviations seldom cause suffering. Uterine and ovarian congestions, inflammations, and neuralgias, are the cause of the pain and the other symptoms. It is, therefore, essential to treat the disease which may be present. Should this have disappeared, local mechanical means may be used.

The majority of patients of the poorer class, who suffer from prolapse, require a prompt and cheap remedy. A pessary here is useful to alleviate the disease; not to cure it. It is, however, necessary always primarily to treat complications, and try to remove them, before its introduction.

Thus, in complete prolapse, we have hypertrophy of the organ; in consequence of displacements of the bladder, we find fungous vegetations; hyperæmia and pain should be treated by leeches and anodynes. Moreover, it is necessary to remove ulcerations thoroughly before permanent retention is thought of.

The only complication in prolapsus which requires no preliminary treatment is hypertrophy of the womb.

The writer but echoes the voice of experienced specialists, when he asserts that the pessary fails in the hands of the inexperienced and the careless. As it emanates from the shop, it is the raw material which has to be adapted to each individual case with the greatest nicety. Those only, who have made its application a study of years, and who have consequently had most success in its use, are entitled to be heard in its praise and condemnation. By far the majority of these adepts have far less confidence in the pessary than formerly, for they remove many more than they insert.

I shall close, with the words of the younger Storer, which fairly represents the voice of the profession: "In many cases, even where skill was used in their adaptation, they did harm. Whilst relieving the backache, difficulty of micturition and inability of walking, it calls the attention of the practitioner from sub-involution, hypertrophy, etc., which had originally occasioned the displacement, and leads the patient to think herself cured, while in reality the disease itself is becoming more chronic.

318 West Madison St.

